# Green synthesis, characterization, and biological evaluation of gold and silver nanoparticles using *Mentha spicata* essential oil

**DOI:** 10.1038/s41598-023-33632-y

**Published:** 2023-05-04

**Authors:** Mir-Hassan Moosavy, Miguel de la Guardia, Ahad Mokhtarzadeh, Seyed Amin Khatibi, Neda Hosseinzadeh, Nasser Hajipour

**Affiliations:** 1 Department of Food Hygiene and Aquatic, Faculty of Veterinary Medicine, University of Tabriz, Tabriz, Iran; 2 Department of Analytical Chemistry, University of Valencia, 50 Dr Moliner Street, Research Building, Burjassot, 46100 Valencia, Spain; 3 Immunology Research Center, Tabriz University of Medical Sciences, Tabriz, Iran; 4 Division of Pharmacology and Toxicology, Department of Basic Sciences, School of Veterinary Medicine, Shiraz University, Shiraz, Iran; 5 Department of Pathobiology, Faculty of Veterinary Medicine, University of Tabriz, Tabriz, Iran

**Keywords:** Biological techniques, Biotechnology, Medical research, Nanoscience and technology

## Abstract

Green synthesis of bioactive nanoparticles (NPs) is getting more attractive in various fields of science including the food industry. This study investigates the green synthesizing and characterization of gold NPs (AuNPs) and silver NPs (AgNPs) produced using *Mentha spicata* L. (*M. spicata*) essential oil as well as their antibacterial, antioxidant, and in vitro cytotoxic effects. The essential oil was mixed with both Chloroauric acid (HAuCl_4_) and aqueous silver nitrate (AgNO_3_) solutions separately and incubated at room temperature for 24 h. The chemical composition of the essential oil was identified by gas chromatography coupled with a mass spectrometer detector (GC–MS). Au and Ag nanoparticles were characterized using UV–Vis spectroscopy, transmission electron microscopy, scanning electron microscopy, dynamic light scattering (DLS), X-ray diffraction (XRD) and Fourier transform infrared (FTIR). The cytotoxicity of both types of nanoparticles was evaluated using MTT assay on cancerous HEPG-2cell line by exposing them to various concentrations of both NPs for 24 h. The antimicrobial effect was evaluated by the well-diffusion technique. The antioxidant effect was determined by DPPH and ABTS tests. According to the results of GC–MS analysis, 18 components were identified, including carvone (78.76%) and limonene (11.50%). UV–visible spectroscopy showed a strong absorption peak of 563 nm and 485 nm, indicating the formation of Au NPs and Ag NPs, respectively. TEM and DLS demonstrated that AuNPs and AgNPs were predominantly spherical shaped with average sizes of 19.61 nm and 24 nm, respectively. FTIR analysis showed that biologically active compounds such as monoterpenes could assist in the formation and stabilization of both types of NPs. Additionally, XRD provided more accurate results, revealing a nano-metal structure. Silver nanoparticles exhibited better antimicrobial activity against the bacteria than AuNPs. Zones of inhibition ranging 9.0–16.0 mm were recorded for the AgNPs, while zones of 8.0–10.33 mm were observed AuNPs. In the ABTS assay, the AuNPs and AgNPs showed a dose-dependent activity and synthesized nanoparticles exhibited higher antioxidant activity than MSEO in both assays. *Mentha spicata* essential oil can be successfully used for the green production of Au NPs and Ag NPs. Both green synthesized NPs show antibacterial, antioxidant, and in vitro cytotoxic activity.

## Introduction

Nanotechnology is considered one of the key technologies of the present century and nanoscience is one of the fast-growing fields of science. Synthesis of metal nanoparticles including gold and silver were developed as they have a wide variety of roles in the improvement of the quality of many industrial, pharmaceutical, and medical products^1^. The color of NPs is dependent on various factors including shape, size, amount of aggregation as well as other properties such as stability, sensitivity, and consistency^1,2^. The synthesized gold and silver nanoparticles are vastly utilized for biomedical applications like treatment against pathogen microorganisms in food science^3,4^. The antimicrobial activity from NPs is principally related to their large surface area, which in causes to greater interaction between the nanoparticles and the cells of the microorganisms. They can exhibit growth inhibition at even low concentrations. Hence, nanoparticles are assumed to the valuable and helpful agents to control the growth of microorganisms in the food industry^4^. NPs can exert cytotoxic effects via multiple approaches including the generation of reactive oxygen and nitrogen species (ROS), interactions with proteins that involve alteration of physicochemical parameters, and as targets for cytotoxicity^5^. Despite many positive features, some potentially toxic and expensive chemicals are utilized as reducing and stabilizing agents in the production process of NPs which restrict the application of chemically produced NPs^6^. Furthermore, the classical approach to the chemical synthesis of NPs has raised concerns for trace amounts of unreacted reagents remain in the solution which contaminates the environment and threatens human health. To overcome these problems, studies started under the green chemistry for seeking benign methods for the synthesis of NPs as well as searching for antibacterial, antioxidant, and anti-cancer effects of natural products. Green way synthesis approaches that employ essential oils and plant extracts as a sources, exhibited a viable substitute to traditional synthesis methods^1^. The composition of these matrices is abundant in many compounds such as phenolic acids, terpenes and flavonoids, which have vital values in medicine. For example, they are good candidates for the synthesis of metal nanoparticles because they can facilitate the reduction of Ag^+^ to Ag^0^ or Au^+^ and Au^3+^ to Au^0^ in the production process of nanoparticles^1,7^. Gold and silver nanoparticles are well-known as fundamental building blocks of nanotechnology. Recently some therapeutic features including anticancer, antiviral, antibacterial, anticancer effect have been related to biogenic Au NPs. High bioavailability, low toxicity and selectivity of Biogenic Au NPs made them a great chemotherapeutic agent for cancer therapy^8^ and antimicrobial agent in the food industry^9^. Many studies have demonstrated the effectiveness of Ag-NPs to inhibit the growth of pathogenic bacteria such as *Staphylococcus aureus*, *Streptococcus mutans*, *Streptococcus pyogenes, Escherichia coli*, and *Proteus vulgaris*^10^. The mechanisms of action by which Ag-NPs exert their antimicrobial effects are not completely clear, but two main hypotheses have been proposed: (i) a direct interaction of the nanoparticle with the cell membrane, and (ii) the release of ionic silver^11^.

Essential oils (EOs) are naturally organic ingredients, both safe and eco-friendly. Nevertheless, the hydrophobic nature of essential oils may be a limiting factor for the synthesis of nanoparticles, for this reason, an aqueous solution of silver salt is used as a silver precursor for synthesis^12^. Accordingly, in-depth studies on the analysis and performance of nanoparticles mediated by essential oils are necessary to confirm the key properties to obtain stable particles.

Essential oils are aromatic and volatile compounds that acquired from different parts of plants including flower, seed, bud, stem, root, leave, wood, stem and bark^13^. One of the main members of the *Lamiaceae* family, is the genus Mentha that defind by 19 species and 13 natural hybrids. For cultivation most popular and usual mint is *M. spicata*. It is particularly grown in temperate regions worldwide, especially in Temperate Asia, Africa, and Europe, however nowadays its cultivation have been done in all over the world^14^. In Iran, utilization of flavoring agent of fresh and dried form of plants and also their essential oils have long been used in several food products, including doogh (Iranian yogurt drink), cheese, beverages, candies, jellies, chocolate, candies, syrups, chewing gum and ice creams. Furthermore, for the treatment in variety of diseases, such as vomiting, nausea, gastrointestinal disorders and also as a breath freshener, toothpaste and antiseptic mouthwash, has been widely applied. The main components of the essential oil obtaind from *M. spicata* are phenolic components such as limonene and carvone^15,16^.

The preparation of silver and gold NPs with *M. spicata* EO has not been studied so far. This oil consists of numerous constituents, containing hydrocarbons and monoterpenes. The key component is Carvone (78.76%); discrete percentages of Limonene, Cis-Dihydrocarveol, and Beta-Bourbonene were also reported^17^. Therefore, the present study aims to investigate the possibility of synthesizing gold nanoparticles and silver nanoparticles in a green way using *M. spicata* essential oil, identifying the synthesized nanoparticles and measuring their cytotoxicity in laboratory conditions in the HEPG-2 cell line and antibacterial activity on common food pathogens and antioxidant effects were measured.

## Results and discussion

### Gas chromatography–mass spectrometry and UV–Vis spectroscopy

**Figure 1:**
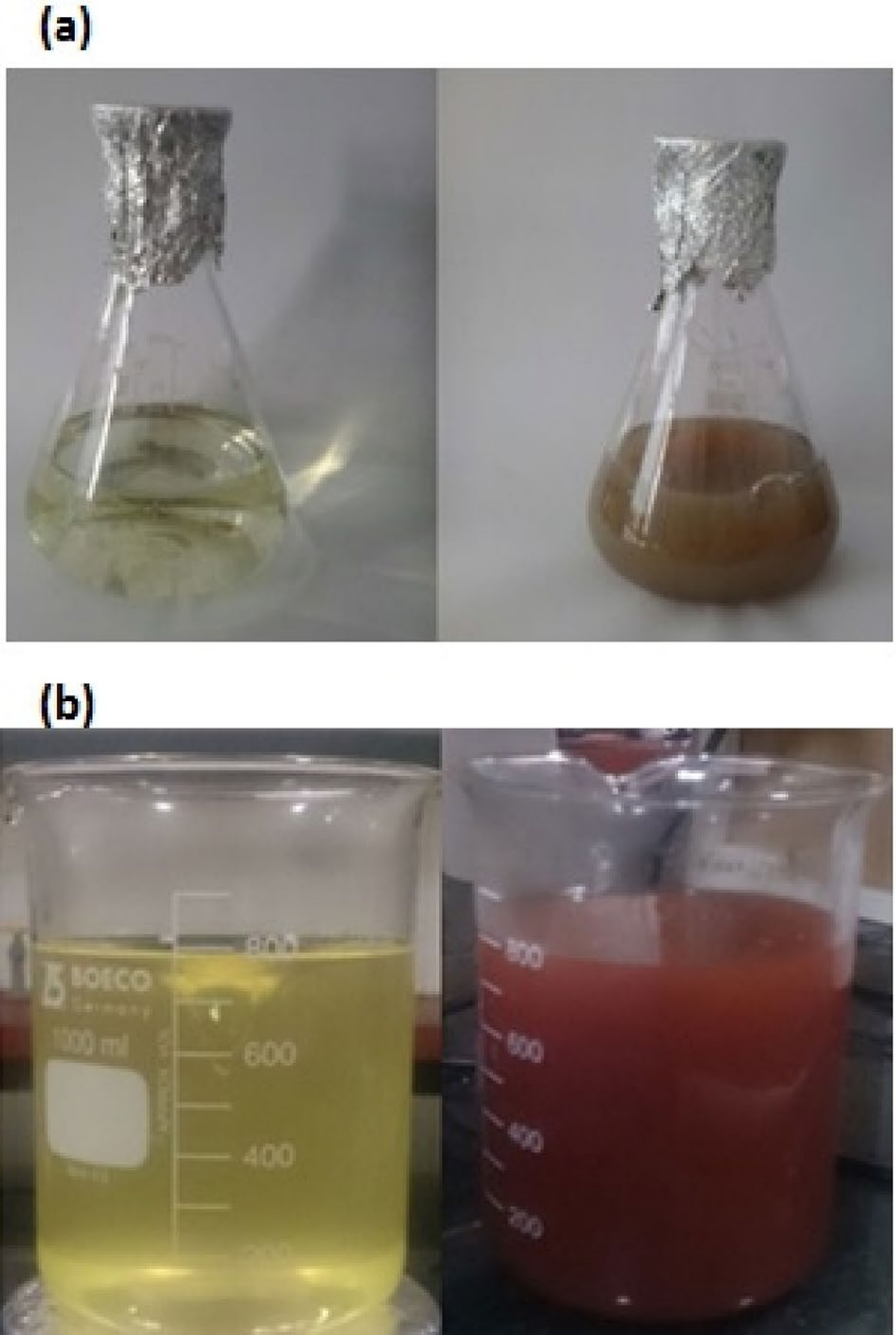
Biosynthesis of AgNPs (**a**) and AuNPs (**b**) by the essential oil of *Mentha spicata* with color change.

In this work, Au NPs and Ag NPs were successfully synthesized by using *Mentha spicata* essential oil as demonstrated by the color change of the reaction medium to ruby-red and light brown, respectively (Fig. 1a,b) which is cause of the effect of surface plasmon resonance and is in agreement with the results of Thanighaiarassu^18^, Muniyappan and Nagarajan^19^, Wang, Xu^20^ and Erci, Cakir-Koc^21^. Previous studies reported that the secondary metabolites such as flavonoids, phenolic acids, tannins, vitamins, proteins, etc. act as reducing and capping agents and are responsible for synthesis of MNPs. The presence of strong reducing phytochemicals in the plant extract not only promotes a fast reduction rate, but also determines the size distribution and morphology of metal nanostructures^22^.

**Figure 2:**
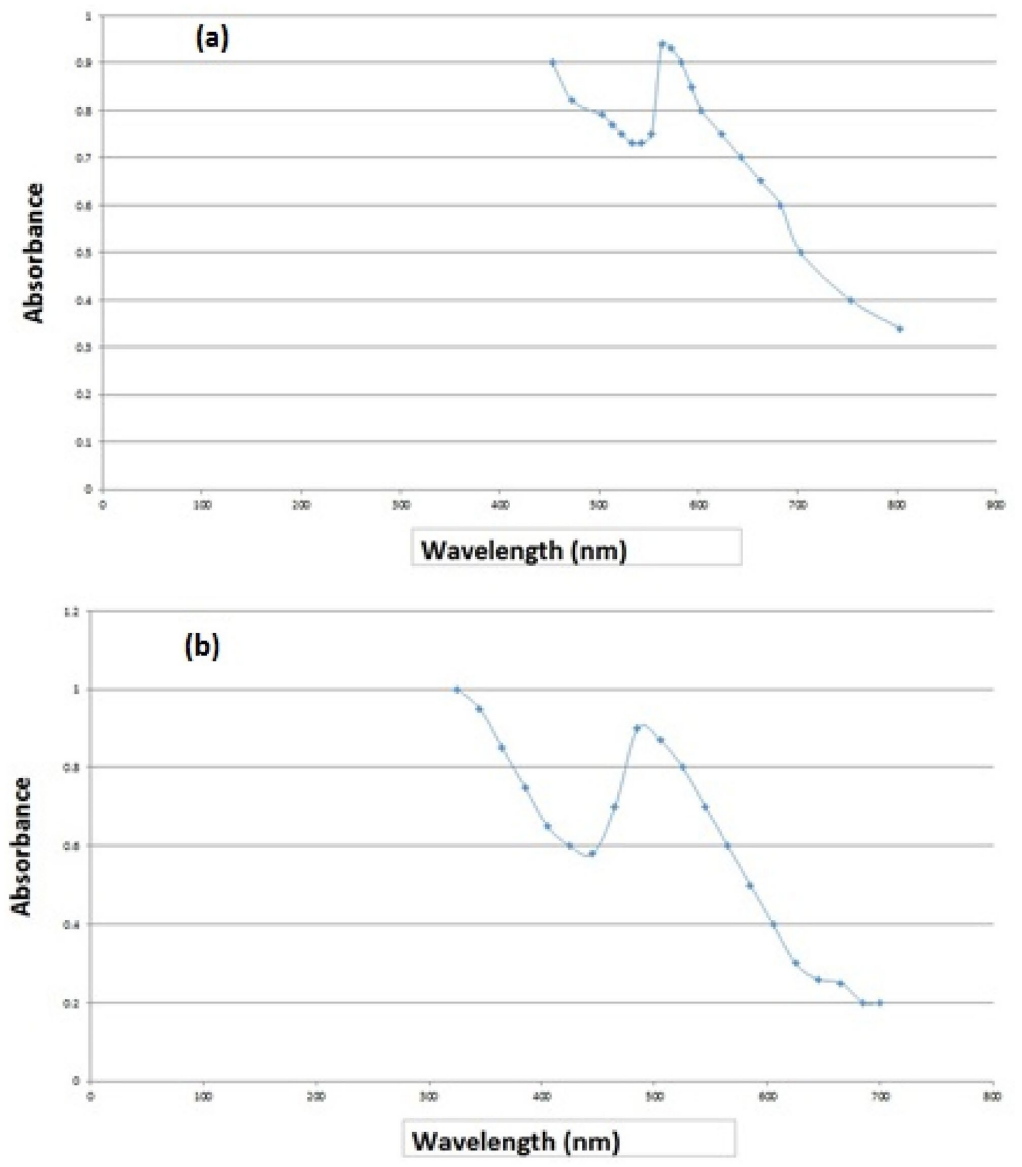
UV–Vis absorption of AuNPs (**a**) and AgNPs (**b**) at different times.

The absorbance of both NPs at different wavelengths was investigated by UV–Vis spectroscopy for 24 h after the addition of essential oil to HAuCl_4_and AgNO_3_solutions. The maximum absorption at 563 nm was seen after 24 h, representing the absorption peak of Au NPs while the highest absorption band was revealed at 485 nm for Ag NPs (Fig. 2a,b). These results were in parallel with the results of Khatami, Soltani Nejad^23^ and Erci, Cakir-Koc^21^. The study of Soltani Nejad^24^ showed the absorption peak of the UV–visible spectrum at 550 nm, which corresponds to triangular gold nanoparticles with a size between 20 and 50 nm. This difference may be the reason for the difference in shape and size range of biosynthesized NPs. In addition, the peak around 400 nm is attributed to the Surface Plasmon Resonance (SPR) which is due to collective oscillations of the conduction of electrons of the NPs^25^.

### TEM analysis

**Figure 3:**
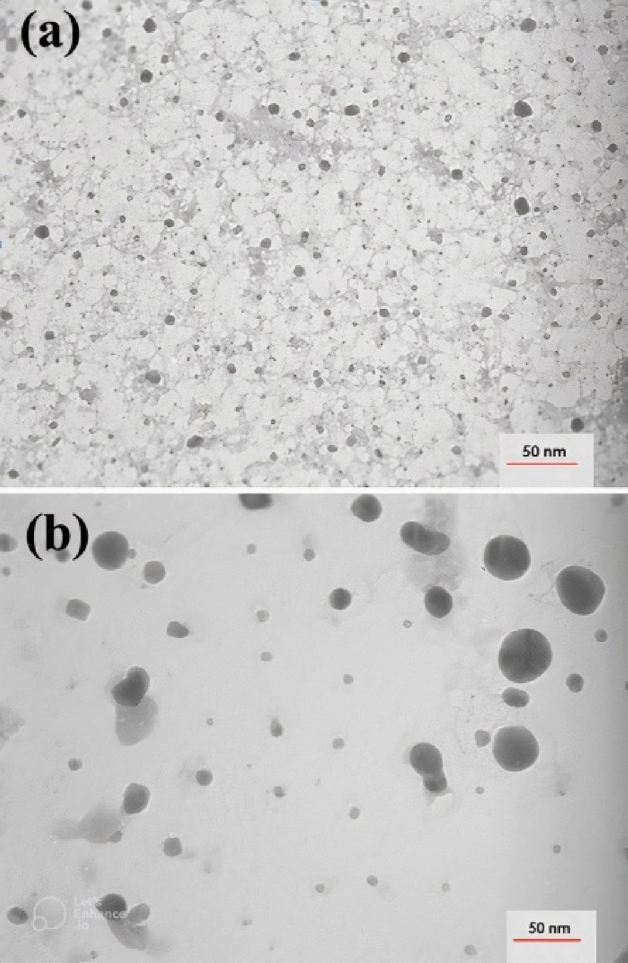
TEM image of synthesized AuNPs (**a**) and AgNPs (**b**) 24 h.

The TEM analysis of the synthesized NPs gives accurate data on the shape, morphology and size of the NPs^26^.The micrographs from the TEM analysis exhibit the spherical shape of the NPs (Fig. 3a,b). In this study, TEM images showed that the NPs are spherical for Au NPs and Ag NPs, respectively.

The calculated average sizes of Au NPs and Ag NPs, detected by the ImageJ (program, ImageJ-win32) were19.61 nm (n = 198) and 24 nm (n = 85), respectively. DLS measurement demonstrated that average size of the NPs were larger than the TEM size, as expected. This size discrimination may reflect the fact that by TEM only the physical size of the nanoparticles is measured, regardless of the coating agent and based on the number of particles, whereas by DLS measurements the hydrodynamic diameter of the nanoparticles is reported to be the same. The diameter of the particle to which the ions or molecules are attached^27^. The ions or other molecules bound to the NPs increase as the size of the NPs increases. As a result, the hydrodynamic diameter of the particles is always larger than the TEM particle size^28^. However, to optimize the size of nanoparticles and their performance in biological experiments, many researchers have shown the importance of the hydrodynamic diameter of the particles^27,29,30^.

### SEM analysis

**Figure 4:**
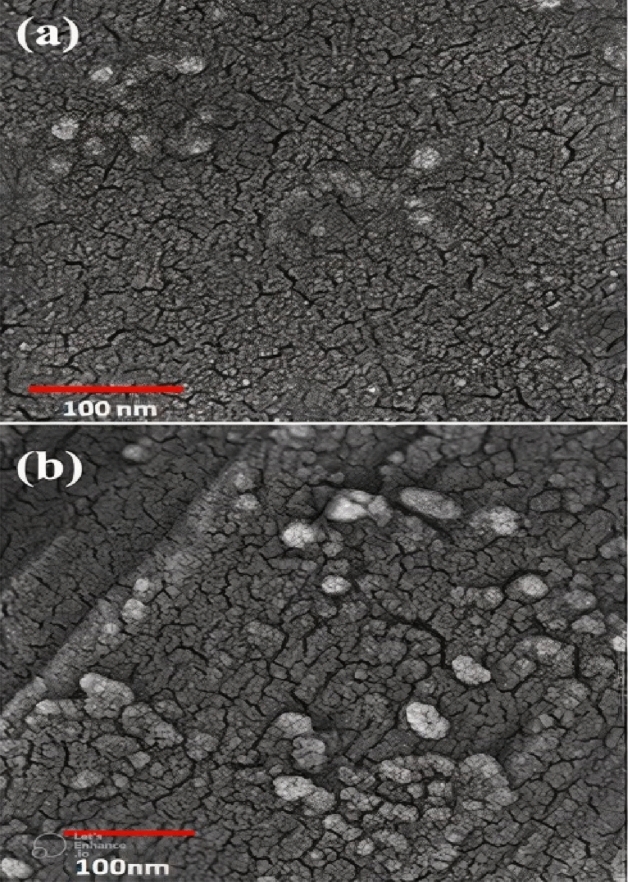
SEM image of silver nanoparticle (**a**) and gold nanoparticle (**b**) synthesized by the essential oil.

In scanning electron microscopy, the morphology of synthesized nanoparticles was approximately spherical shaped (Fig. 4a,b).

### XRD analysis

**Figure 5:**
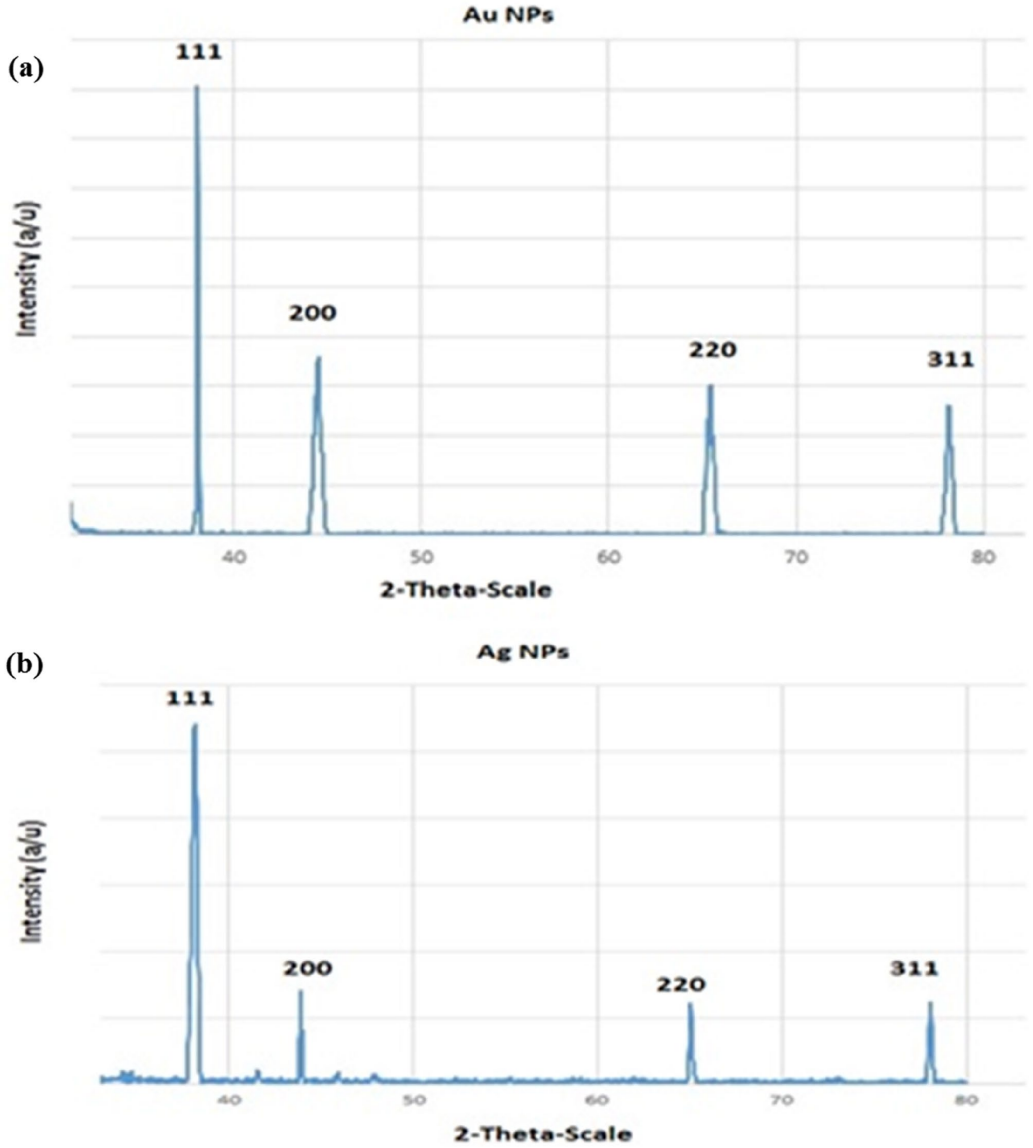
XRD pattern of biosynthesized AuNPs (**a**) and AgNPs (**b**).

The XRD analysis was carried out to verifying the crystalline nature of the as obtained NPs using an X-ray diffraction method Bennur^30^. Figure 5a,b display the XRD pattern of the prepared NPs. Four peaks at 2θ values of 38.2°, 44.5°, 64.8°, and 78° corresponding to (111), (200), (220), and (311) planes of gold and silver were observed. In the present study, the XRD pattern was similar to the one taken in the results of the study done by Khatami^23^.

### FTIR spectroscopy analysis

**Figure 6:**
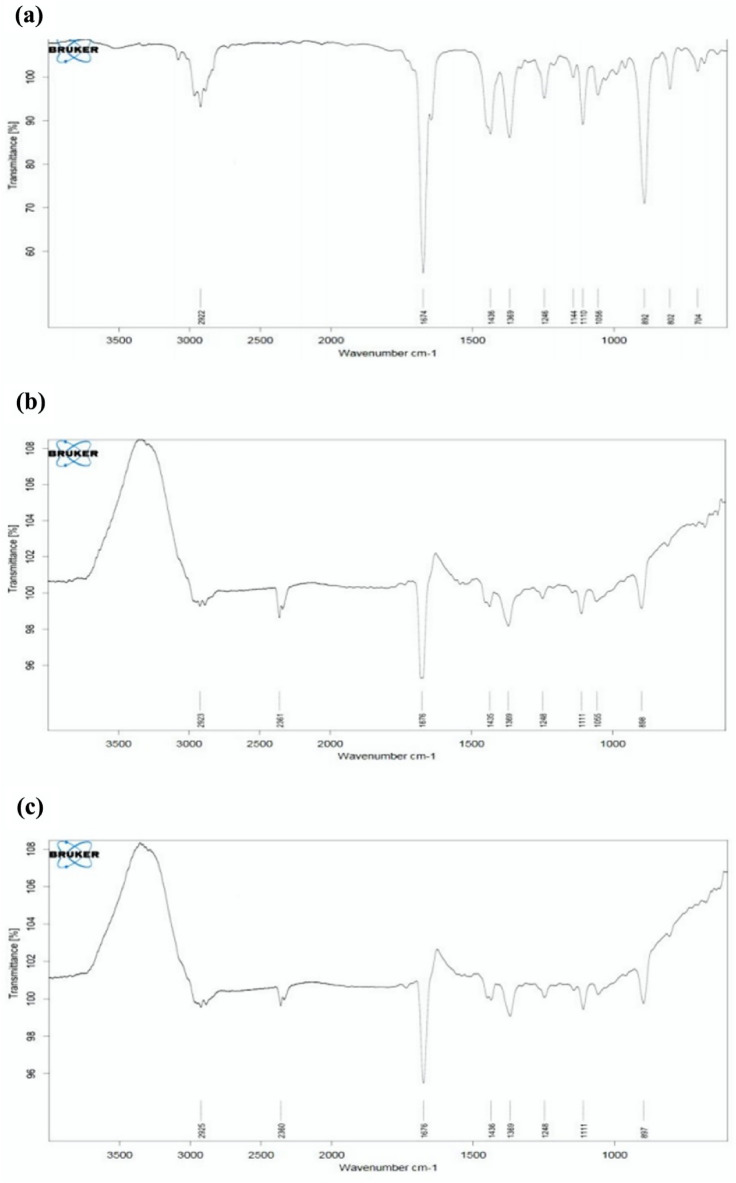
The FTIR spectrums of crude essential oil (EO) (**a**), the biosynthesized EO-AuNPs (**b**) and the biosynthesized EO-AgNPs (**c**).

The evaluation of the presence of likely biomolecules on the surface of the as prepared NPs can be evaluated by FTIR analysis^31^. In this work, FTIR spectroscopic studies were carried out to identify possible reducing agents present in essential oil. As shown in Fig. 6a, strong bands at 2922, 1674, 1436, 1369, 1246, 1144, 1110, 1056, 892, 802 and 704 cm^−1^ were observed for mint essential oil, while at 2955, 2360, 1436, 1369, 1248, 1111, and 897 cm^−1^ for biosynthesized AuNPs. As shown in Fig. 6b,c, the peak observed at 2955 is characteristic of the C–H group that exists in monoterpenes structures such as limonene and carvone which can be responsible for reducing and covering gold and silver ions^32,33^. The appearance of the band at 2360 cm^−1^ can be assigned to the triple bond C≡C presents in the AuNPs. There is a peak at 1676 cm^−1^ which is indicative of the presence of a stretch C=O bond in some constituents. The lack of a peak at 1144 cm^−1^ demonstrate that stretch C–O groups are involved in the synthesis of AuNPs. After the decline of gold and biosynthesis of AuNPs, the peaks at 1246, 1110, and 892 cm^−1^ have been shifted to 1248, 1111, and 897 cm^−1^, respectively. Comparing the FTIR spectrum of AuNPs with essential oil, the presence of compounds containing bands like C–H, C≡C, and C–O responsible for reducing and capping the silver ions.

FTIR spectroscopic studies were also carried out for AgNPs and essential oil to identify possible reducing agents. As shown in Fig. 6a, strong bands at 2922, 1674, 1436, 1369, 1246, 1144, 1110, 1056, 892, 802 and 704 cm^−1^ were observed for mint essential oil, while at 2923, 2361, 1676, 1435, 1369, 1248, 1111, 1035, and 898 cm^−1^ for biosynthesized AgNPs. As shown in Fig. 6b, the peak observed at 2923 is characteristic of the C–H group that exists in monoterpenes structures such as limonene and carvone. The appearance of the band at 2361 cm^−1^ can be assigned to the triple bond C≡C presents in the AgNPs. There is a peak at 1676 cm^−1^ which is indicative of the presence of a stretch C=O bond in some constituents. The absence of a peak at 1144 cm^−1^ indicates that stretch C–O bonds are involved in the synthesis of AgNPs. After reducing silver and biosynthesis of AgNPs, the peaks at 1436, 1246, 1110 and 892 cm^−1^ shifted to 1435, 1248, 1111, and 898 cm^−1^, respectively. The comparison of the FTIR spectrum of the AgNPs with the essential oil revealed the presence of compounds containing the bands such as C–H, C≡C, and C–O responsible for reducing and capping the silver ions.

### DLS analysis

**Figure 7:**
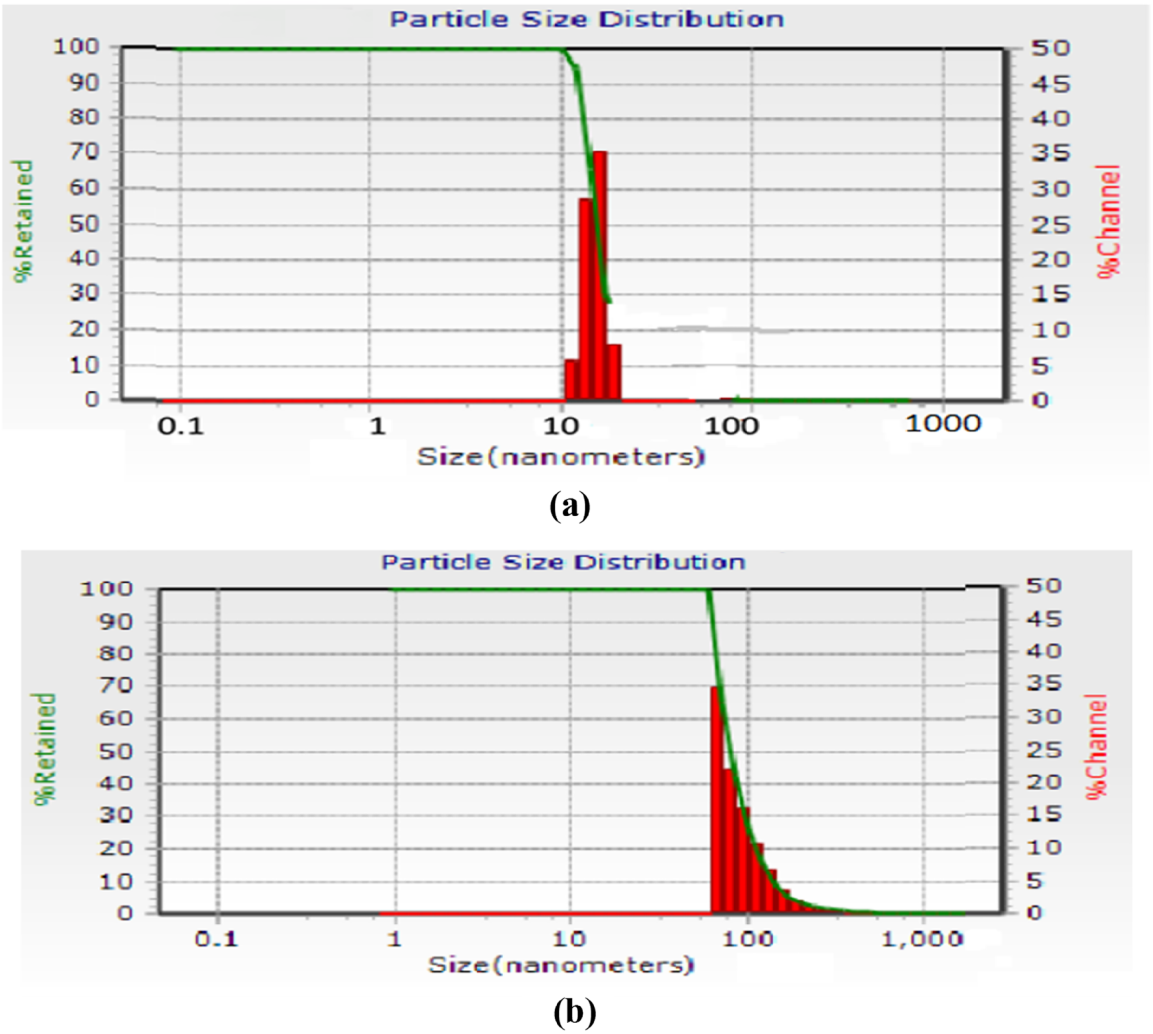
DLS pattern of biosynthesized AuNPs (**a**) and AgNPs (**b**).

Twenty-four hours after adding both solutions to the essential oil, DLS analysis was used to analyze the particle size distribution. The AuNPs and AgNPs size range show that they have a non-uniform distribution, with an average particle size of 47.24 nm and 72.38 nm, respectively (Fig. 7a,b).

### MTT assay

**Figure 8:**
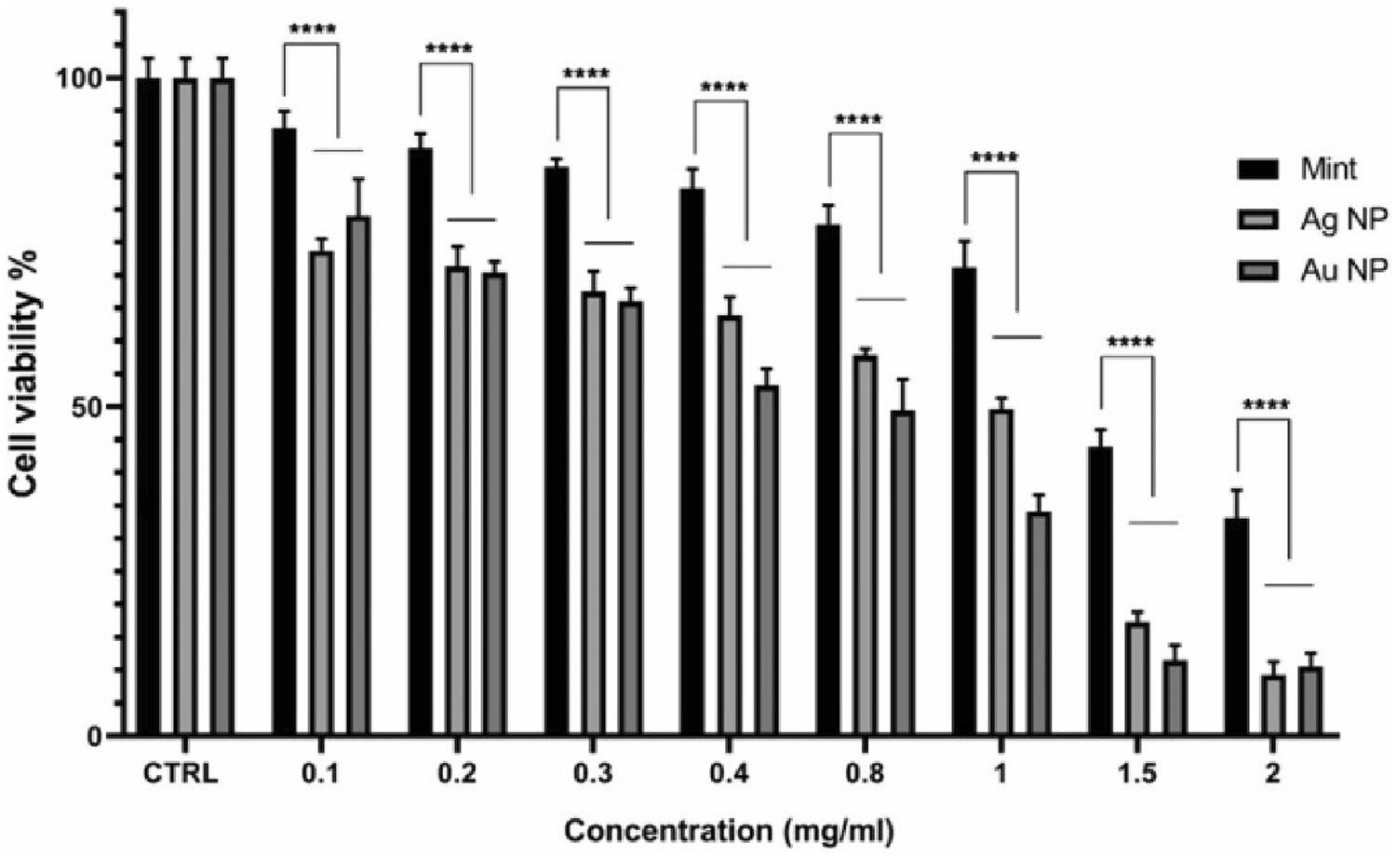
The effects of AuNPs and AgNPs on the viability of HEPG-2 cells.

**Figure 9:**
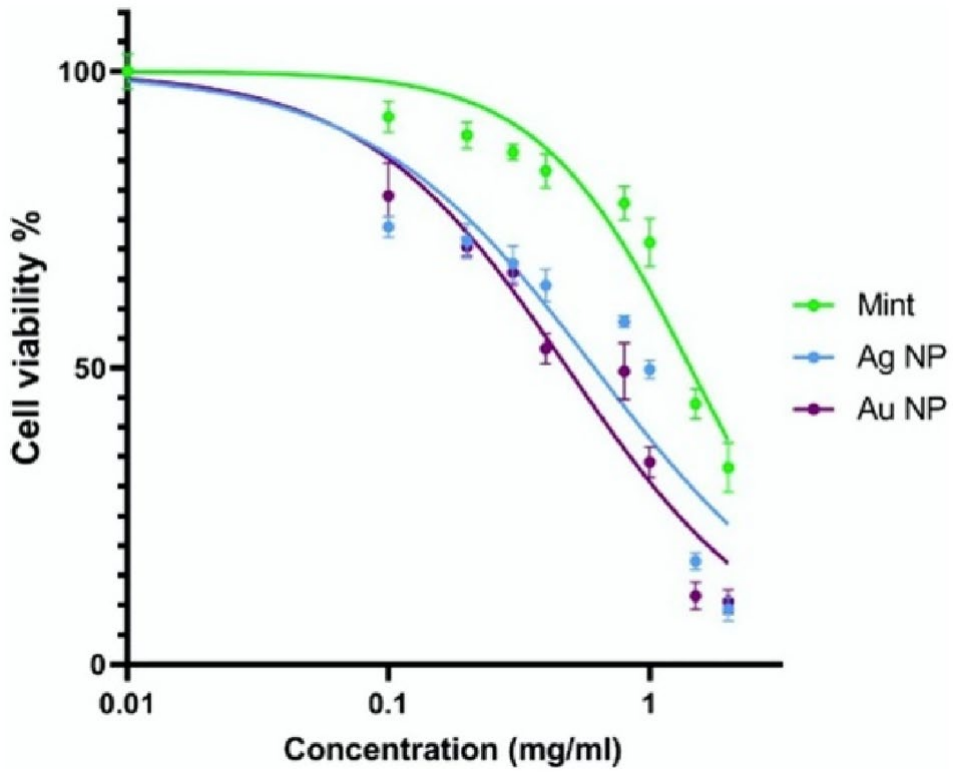
Cytotoxicity of AuNPs and AgNPs on HEPG-2 cell line. The sensitivity of HEPG-2 cells to AuNPs was more than AgNPs.

The cytotoxicity of NPs could be affected by functional groups, surface charge and nanoparticle size. Furthermore, cell type play as a determinant factor in sensitivity to cytotoxic effects^34^. The effect of biosynthesized AuNPs and AgNPs on HEPG-2 cells was assessed by MTT assay, which is shown in Fig. 8. The green synthesized NPs, both represent in-vitro cytotoxic property against HEPG-2 cells. The higher concentrations of both NPs (0.1–2 mg/mL) significantly lower the viability of cells in a dose-dependent manner (Fig. 8). HEPG-2 cell line showed greater sensitivity to AuNPs (IC50 = 0.4834 mg/mL) than AgNPs (IC50 = 0.6145 mg/mL) after exposure for 24 h. (Fig. 9).

There are three proposed mechanisms for the anticancer activity of biological NPs. Firstly, the apoptotic pathway, which depends on an increased level of ROS which leads to oxidative stress and DNA fragmentation in the cancerous cell. Secondly, interference of proteins/DNA, resulting in cell chemistry functions. Thirdly, the interaction of biological NPs to cell membranes makes changes in the cell permeability and mitochondrial dysfunction. It has been revealed that the activation of p38 MAPK and Caspase-3 at gene and protein expression levels results in response to nanoparticles^35^.

### Antibacterial activity

The antimicrobial activity of the prepared gold and silver nanoparticles was evaluated using five different bacteria, including *E. coli, L. monocytogenes*, S. *Typhimurium*, *S. aureus,* and *B. cereus.* Tables 1 and 2 show that with the increase of the nanoparticles, the diameter of the inhibitory zone increased dramatically. Silver nanoparticles exhibited better antimicrobial activity against the bacteria than AuNPs. Zones of inhibition ranging 9.0–16.0 mm were recorded for the AgNPs, while zones of 8.0–10.33 mm were observed AuNPs. *B. cereus* had the highest resistance against gold nanoparticles.

**Table 1:** Diameter of inhibition zone (mm) produced by silver nanoparticles (AgNPs) against various bacteria. Values are Mean ± SD (n = 3).

Bacterial strain	100 µg/mL	50 µg/mL	25 µg/mL	12.5 µg/mL
*E. coli*	16.0 ± 0.0^a^	15.33 ± 0.57^a^	14.33 ± 1.15^a^	13.33 ± 1.15^a^
*S. Typhimurium*	12.33 ± 0.57^b^	11.66 ± 0.57^b^	11.00 ± 0.0^b^	9.66 ± 0.57^b^
*L. monocytogenes*	15.33 ± 0.15^a^	14.66 ± 1.52^a^	14.00 ± 1.00^a^	14.16 ± 1.04^a^
*S. aurerus*	12.66 ± 0.57^b^	11.66 ± 0.57^b^	10.66 ± 0.57^b^	9.66 ± 0.57^b^
*B. cereus*	14.00 ± 1.00^ab^	13.33 ± 1.30^ab^	11.00 ± 0.00^b^	9.00 ± 0.00^b^

The different letters (a–b) indicate a significant difference (P < 0.05) among various bacteria.

**Table 2:** Diameter of Inhibition zone (mm) produced by gold nanoparticles (AuNPs) towards various bacteria. Values are Mean ± SD (n = 3).

Bacterial strain	100 µg/mL	50 µg/mL	25 µg/mL	12.5 µg/mL
*E. coli*	10.33 ± 0.57^a^	10.00 ± 0.00^a^	8.66 ± 0.57^a^	ND*
*S. Typhimurium*	9.00 ± 0.00^bc^	08.66 ± 0.57^b^	8.33 ± 0.57^a^	ND
*L. monocytogenes*	9.66 ± 0.57^ab^	8.66 ± 0.57^b^	ND	ND
*S. aureus*	8.66 ± 0.57^ cd^	8.00 ± 0.00^b^	ND	ND
*B. cereus*	8.00 ± 0.00^d^	ND	ND	ND

*Not detected.

The different letters (a–b) indicate a significant difference (P < 0.05) among various bacteria.

It was noted that AuNPs and AgNPs exhibited superior antibacterial activity against *E. coli*, inducing a greater inhibition compared to other bacteria. Similar results have been reported by Oueslati and Ben Tahar^36^ and Vilas^37^.

AuNPs in the concentrations of 12.5 µg/mL did not show any inhibitory zone around the studied bacteria. The largest inhibition was observed by silver nanoparticles against *E. coli*.

The antimicrobial effect of Silver has long been known in medical and industrial processes. One of The most important applications of silver and silver nanoparticles in medicine is in topical ointments to prevent infection of burns and open wounds^38^. The other outcomes of silver nanoparticles is cancer treatment that is in relationship with their fabulous properties such as alteration in metabolic activity, inducing of reactive oxygen species (ROS) and gene alteration in cancer cells^39^.

The antibacterial action of silver nanoparticles can be related to the reaction of metal ions with thiol and sulfhydryl groups in the bacterial cell membrane protein. This reaction can reduce the permeability of the bacterial cell leading to destruction of cellular respiration and, consequently, cell death. Besides, Ag can bind to bacterial DNA, causing denaturation and inhibiting replication^4^. Due to containing great number of silver atoms in silver nanoparticles they can act as an antibacterial substance in both gram positive and gram negative bacterias by penetrating into the pathogen’s cells and led to DNA gyrase blocking^40^.

In accordance with the findings of niloufar work, antibacterial activity of AgNPs can be attributed to the ROS (reactive oxygen species) of bacterial cell. Accordingly AgNPs could harm DNA and membrane proteins by enforcing the release of ROS. Generally, antibacterial activity of silver nanoparticles have been reported previously and demonstrated that silver nanostructures can cause cell damage by accumulating in cell cytoplasm, following the coagulation with sulfur and phosphorus containing compounds in order to deactivate the intracellular enzymes. These functionalities of silver nanoparticles can be contributed in bacterial cell lysis by coagulation, due to owning of sulfur and phosphorus in bacteria’s cell membrane, DNA and proteins of the membrane^41^.

Also, AuNPs have antimicrobial activity through binding to the surface of microorganisms, causing damage to the flagella and destruction of the cell walls^42^.

Gold nanoparticles exhibited anticancer activity through colorectal cancer cells by various mechanisms that led to cell death. In addition, Barabadi revealed that AuNPs express potential anticancer activity against cervical cancer cells^43^.

As reported in Barabadi et al.^22^, study, the AgNPs that synthesized via green method demonstrated higher antibacterial activity in compromise with chemically synthesized AgNPs.

In Gram-positive bacteria (*L. monocytogenes*, *S. aureus,* and *B. cereus)*, the protecting effect of the peptidoglycan layer on the antibacterial activity of nanoparticles resulted in lower inhibition zones compared to *E. coli*^38^. An exceptionally lower inhibition zone was observed by Gram-negative bacteria, S. *Typhimurium*.

The antibacterial activity of metallic NPs can be explained by several mechanisms. One of the main mechanisms for their bactericidal action is the oxidative dissolution of these particles. In an oxygenated medium, nanomaterials act as reservoirs of metallic ions which turn into a potential antibacterial agent. Furthermore, the components of essential oil affect the antibacterial activity of the synthesized nanoparticles by damaging the bacterial cell membrane^36^.

The results of this study provide an important basis for the application of silver and gold nanoparticles produced using the essential oil of *M. spicata* in the treatment of human infections associated with the microorganisms used in this study.

### Antioxidant effect

The antioxidant activity of AuNPs and AgNPs was investigated using ascorbic acid and BHT (positive controls) against DPPH and ABTS radicals at different concentrations ranging from 12.5 to 100 μg/mL. The result shows a significant decrease in the concentration of DPPH and ABTS radicals due to the scavenging activity of MSEO, AuNPs, AgNPs, and standard.

**Figure 10:**
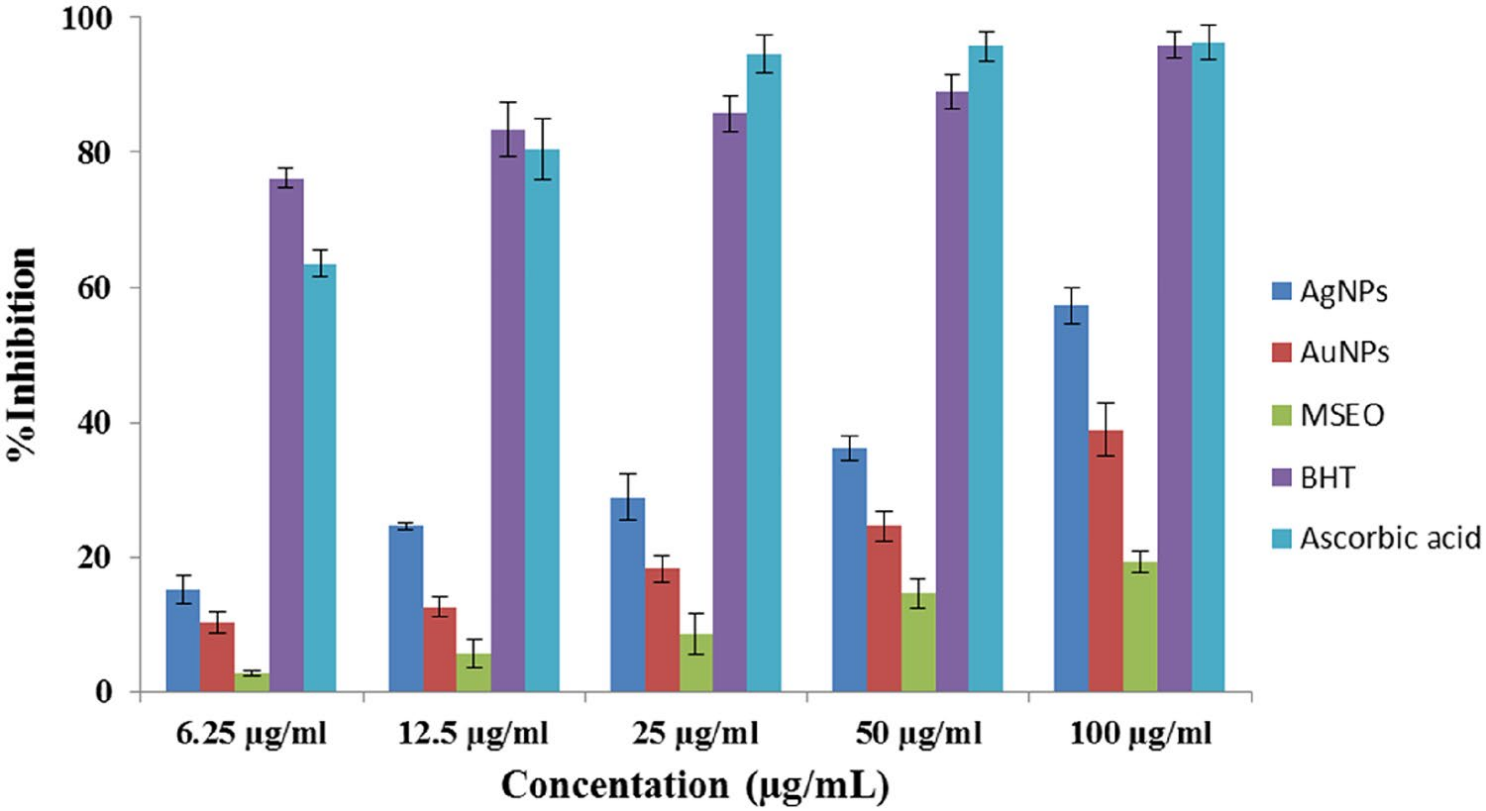
DPPH radical scavenging activities of silver nanoparticles (AgNPs), gold nanoparticles (AuNPs), *Mentha spicata* essential oil (MSEO), Butylated hydroxytoluene (BHT), Ascorbic acid.

Figure 10 shows that the DPPH radical scavenging activity was increased with increasing of NPs concentration. This result is in good agreement with the previous reports in the literature^21,44–47^. The highest inhibition of 38.90% and 57.20% was observed at 100 μg/mL by AuNPs and AgNPs, respectively.

**Figure 11:**
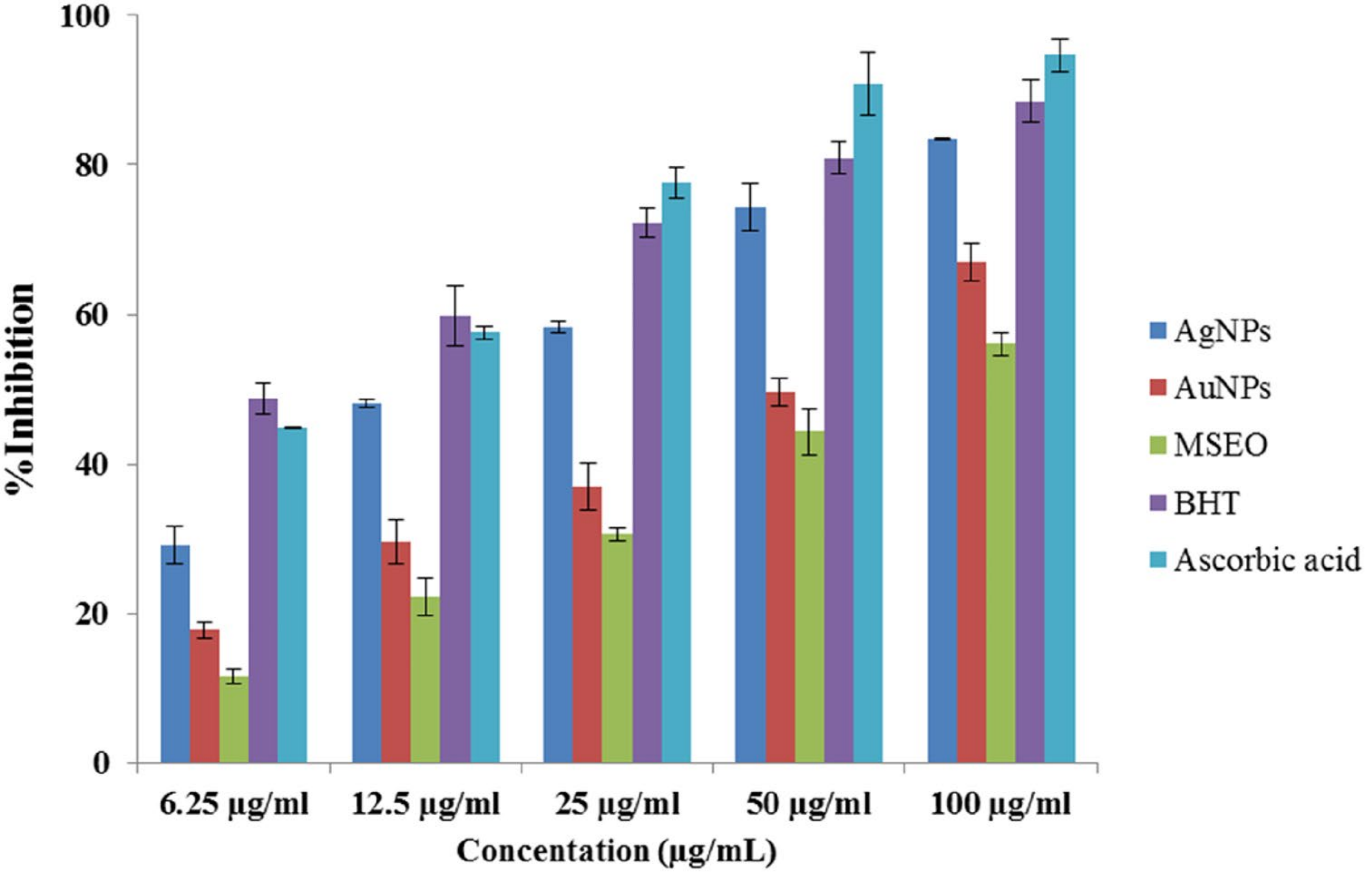
ABTS radical scavenging activities of silver nanoparticles (AgNPs), gold nanoparticles (AuNPs), *Mentha spicata* essential oil (MSEO), Butylatedhydroxytoluene (BHT), Ascorbic acid.

The antioxidant capacity of AuNPs and AgNPs was further evaluated by ABTS scavenging. In the ABTS assay, the AuNPs and AgNPsalso showed a dose-dependent activity (Fig. 11). Synthesized nanoparticles exhibited higher antioxidant activity than MSEO in both assays. These results suggest the good antioxidant activity of the synthesized nanoparticles. Also, AgNPs had superior activity to AuNPs in both tests. The difference in the attached functional groups of MSEO to nanoparticles may be responsible for the differences in their scavenging activity. These results agreed with the previous result reported by Adebayo^44^ where the antioxidant activity of the synthesized gold and silver nanoparticles from *Opuntia ficus-indica* extract was evaluated using Nitric Oxide (NO). Higher DPPH radical scavenging activity was also reported By Huo^45^ in silver chloride nanoparticles of *Glycyrrhiza uralensis* (Gu-AgClNPs) when compared to gold nanoparticles of *Glycyrrhiza uralensis* (Gu-AuNPs). Surface morphology could affect the biological activity of nanoparticles. In a study spherical shaped of synthesized silver nanoparticles represented high antioxidant activity and that was in agreement with our findings^41^. The antioxidant activity of the prepared nanoparticles has been observed to be a result of the absorption or integration of more bioactive compounds or bioreductant molecules of the plant on the surface of the nanoparticles which increases the surface area for radical scavenging activity (Fig. 10). Hence, the reducing effect of the nanoparticles can be attributed to the phenolic functional groups on the surface of nanoparticles^44^.

## Conclusion

In this study, a low-cost and simple method was used to synthesize AgNPs and AuNPs using essential oil from *Mentha spicata* through the reduction of Ag^+^ and Au^3+^ ions. This might be due to the presence of phenolic and flavonoid compounds in essential oil as electron donors. The formation of AgNPs and AuNPs was confirmed using UV–Vis Spectroscopy, dynamic light scattering, SEM, TEM, FT-IR, and XRD.The micrographs from SEM and TEM analysis proved the formation of small size and spherical shape of the nanoparticles. FTIR analysis showed that biologically active compounds such as monoterpenes could assist in the formation and stabilization of both types of nanoparticles. XRD analysis showed nano-metal structures in particles. The synthesized nanoparticle exhibited a strong cytotoxic, antibacterial and antioxidant activity. The AgNPs showed higher antioxidant and antimicrobial activities than AuNPs. However, the cytotoxicity study showed that the HEPG-2 cell line was more sensitive to AuNPs than AgNPs after 24 h of exposure. The procedure for the biosynthesis of nanoparticles has several advantages such as cost-effectiveness, compatibility for biomedical and pharmaceutical, and food applications as well as for large-scale commercial production. In the future, it would be significant to select such plants' EO compounds, to understand the clear mechanism of biosynthesis, and to technologically improve the nanoparticles to achieve better control oversize, shape, and absolute mono dispersivity to utilize the potential of herbal medicine in nanoscience for biomedical applications.

## Methods

### Essential oil

The commercial *Mentha spicata* EO used in this study was purchased from Barij Essence Co. Ltd. (Kashan, Iran). The plant essential oil was kept at a temperature of 4–6 °C before analysis. The essential oil was used as a reducing agent as well as a stabilizing agent for producing NPs.

### Gas chromatography: mass spectrometry

The gas chromatography-mass spectrometry (GC–MS) was accomplished using an Agilent 7890/5975C GC–MS system, fitted with a DB-624 capillary column (30 m length × 0.25 mm; 0.25 µm film thickness) for analysis of the essential oil. The column temperature program was adjusted as follows: the initial temperature of the oven was set to 60 °C and held at this temperature for 3 min. The temperature was then increased to 220 °C at a rate of 5 °C per min. and held for 1 min. The temperature of the injector was 250 °C. The amount of injection was 0.2 μl. The carrier gas was Helium with a flow rate of 1.2 mL/min and a split ratio equal to 1:4. The mass spectrometer was operated in EI ionization mode at 70 eV, and complete scans from 40 to 350amu (atomic mass units) were recorded. The constituents of the essential oil were identified by the confirmation of the experimental gas chromatographic retention indices (RI) relative to n-alkanes (C8–C24) and mass fragmentation with those of the National Institute of Standards and Technology (NIST 08) commercial library, as well as with literature data^46^. All analyses were carried out in triplicate.

### Green synthesis of nanoparticles

Chloroauric acid (HAuCl_4_) and silver nitrate (AgNO_3_) purchased from Sigma-Aldrich, Germany was used in the test. Thus, 0.5 mL of the essential oil was mixed with 19 mL of HAuCl_4_ and AgNO3 solutions separately (10 mmol/mL) in a conical flask and then a magnetic stirrer is kept inside the conical flask and started to run vigorously on the hot plate. They were incubated at room temperature for 24 h. The reduction of gold ions was initially confirmed by visual inspection of color change from pale yellow to ruby-red (AuNPs) and light brown (AgNPs) Then by UV–visible spectrophotometer at different times (0, 30 min, 2 h, 4 h and 24 h). To separate the unreacted essential oil, the sample was centrifuged at 6000 rpm for 11 min. The sediment left at the end of the tube was resuspended in distilled water. The water was evaporated in a furnace. The thin layer of NPs remaining on the watch glass surface was used for further studies^18^.

### Characterization of NPs

UV–visible spectrophotometer (Analytik Jena, Specord 250 Plus, Germany) was used for the characterization of NPs. Maximal absorption was scanned at the wavelength of 200–800 nm. Dynamic light scattering (DLS) analysis (Microtrac, Nanotrac wave II, USA) was used to estimate the average size of nanoparticles in the initial stage. Particle size and shape of nanoparticles were observed using transmission electron microscope (TEM) (LEO, 906E, Germany) and a scanning electron microscope (Tescan Mira, Czech republic). X-ray diffraction (XRD) analysis of the NPs drop-coated on glass was done on TD-3700, Tongda X-Ray Diffractometer (Dandong Tongda science and technology, China) operatingat a voltage of 40 kV and a current of 20 mA with Cu Kα radiation. The instrument was operated over the 2θ range of 20–80 °C. Fourier transform infrared (FTIR) spectral analysis was carried out to identify the possible biomolecules responsible for synthesizing NPs. The spectra of essential oil were recorded before and after adding chloroauric acid and silver nitrate solutions. The spectrum was recorded in the range of 500–4000 cm^−1^ (Bruker, Tensor 27, Germany).

### Cell lines and culture conditions

The HEPG-2 cell line was acquired from the National Cell Bank of Iran (Pasteur Institute, Tehran, Iran) and cultured in 75 cm^2^ culture flasks using DMEM medium supplemented with 10% heat-inactivated fetal bovine serum (Gibco Inc., USA) and streptomycin-penicillin (100 µg/mL and 100 IU/mL, respectively) in an incubator providing 5% CO_2_and 95% humidity at 37 °C. Every 2 days culture medium was replaced with fresh medium until reaching suitable confluency of about 70%. Then they were detached with Gibco Trypsin–EDTA (0.25%) and subcultivated.

### MTT assay

The density of viable cells was determined by Trypan blue exclusion assay by using a hemocytometer and the preparation was diluted with the DMEM medium to yield optimal plating densities for cells^47^ before the MTT assay.HEPG-2 cells were seeded in a 96-well flat bottom microtiter plate at a density of 12 × 10^3^ cells/well and allowed to adhere. After 48 h of incubation and producing the confluent cells, the stock solution of AuNPs and AgNPs was freshly prepared and diluted with the cell culture medium to the desired concentrations (0.1–2 mg/mL). After 24 h of exposure, the cells without treatment were used as control and received only the culture medium as the final concentration. The cytotoxicity test was carried out by MTT assay according to the manual of the manufacturer for investigating changes in mitochondrial/non-mitochondrial dehydrogenase activity. 50 µl of 2 mg/mL MTT solution (Sigma Aldrich, Germany) was added to each well and the plate was incubated for 4 h at 37 °C in a CO_2_ incubator. The medium was then aspirated, and the formed formazan crystals were solubilized by adding 100 μl of DMSO per well for 10 min at 37 °C in a CO_2_ incubator. Following half an hour, optical density (OD) was defined at 570 nm in a microplate reader (Tecan, Switzerland). Cell viability was expressed based on the absorbance of the control wells, which were considered as 100% of absorbance. Cytotoxicity was expressed as the concentration of the substance (both AuNPs and AgNPs) inhibiting cell growth by 50% (IC50). All reactions were performed in triplicate. The following equation was used to determine the percent of cell viability^20^.

$$ \% {\text{Viability}} = \left( {{\text{OD}}\,{\text{treated}}\,{\text{cells}}/{\text{OD}}\,{\text{control}}\,{\text{cells}}} \right) \times {1}00 $$

### Antibacterial activity

*Escherichia coli, Listeria monocytogenes*, Salmonella *Typhimurium*, *Staphylococcus aureus,* and *Bacillus cereus* were prepared in a lyophilized form by the Iranian Research Organization for Science and Technology (IROST, Karaj, Iran). The bacteria were transferred from a sterile microtube to brain heart infusion (BHI) agar (Merck, Germany) and incubated at 37 °C for 24 h. The second culture was prepared from the first culture and incubated under the same condition. Serial dilutions were prepared from the bacterial culture using the tubes containing 9 mL of sterile peptone water (0.1%, v/w). Then, 0.1 mL of each dilution was cultured on the surface of Muller Hinton agar (Merck, Germany) to determine the bacterial concentration.

The antimicrobial effect of nanoparticles was studied against the selected bacteria by well diffusion technique. First, the bacterial suspension (with a final concentration of 10^5^ CFU/mL) was cultured on the surface of Muller Hinton agar. Then, wells with a diameter of 6 mm were created on the culture medium. 50 µL of synthesized nanoparticles in different concentrations (100, 50, 25, 12.5 µg/mL) were transferred into the wells under sterile conditions. After incubating the plates at 37 °C for 24 h, the diameter of the inhibition zone around the wells was measured.

### Antioxidant effect

The antioxidant effect of nanoparticles to inhibit was evaluated using DPPH (2, 2-diphenyl-1-picrylhydrazyl) radical as described by Maciel^48^. For this purpose, an aliquot of 1.5 mL different concentrations of nanoparticles (100, 50, 25, 12.5 and 6.25 µg/mL) was mixed with 1.5 mL of DPPH solution (0.2 mmol L^−1^). Then, the above mixture was completely homogenized and incubated at 24 °C in the dark for 30 min. The absorbance of the mixture was measured at 520 nm. Butylated hydroxytoluene (BHT) and ascorbic acid will be used as a standard.

To evaluate the scavenging activity of nanoparticles, the ABTS^•+^ assay was also used according to the method described by Bakur^49^. The free radicals of ABTS^•+^ were prepared by mixing the stock solution of ABTS (2, 2′-azino-bis (3-ethylbenzothiazoline-6-sulfonic acid)) (0.7 μM) and potassium persulfate (2.45 μM). The mixture was incubated in a dark environment at room temperature for 16 h. The solution containing ABTS^•+^ free radicals was diluted with 80% ethanol until its absorbance reached 0.700 ± (0.05) at 743 nm. Then, 200 µL of ABTS^•+^ solution was mixed with 20 µL of nanoparticles at different concentrations (100, 50, 25, 12.5, and 6.25 µg/mL). After incubation for 6 min in a dark place, the absorbance of the samples was measured at 734 nm using ascorbic acid and BHT as standard. Antioxidant activity of DPPH and ABTS^•+^ radicals by AgNPs and AuNPs were calculated according to the following formula:

$${\rm Antioxidant\,activity} ({\%}) = 100-\left[\frac{\left({{\rm Abs}}_{{\rm sample}}-{{\rm Abs}}_{{\rm blank}}\right)\times 100 }{{{\rm Abs}}_{{\rm control}}} \right]$$

where Abs_sample_is the absorbance of sample/standard mixed with DPPH radical in methanol; Abs_blank_is the absorbance of methanol and Abs_control_ is the absorbance of DPPH radical in methanol; Abs_blank_is the absorbance of methanol.

### Statistical analysis

Data were expressed as mean ± standard deviation (SD) of three independent tests. The statistical difference in data was evaluated using independent t-test and one-way ANOVA test using SPSS software version 19.0. *P* values < 0.05 was considered statistically significant.

### Statement of compliance

This experimental research on plants complies with relevant institutional, national and international guidelines and legislation.

## Data Availability

All data used to support the conclusions of this study are included within the article.
